# ABCC4/MRP4: a MYCN-regulated transporter and potential therapeutic target in neuroblastoma

**DOI:** 10.3389/fonc.2012.00178

**Published:** 2012-12-19

**Authors:** Tony Huynh, Murray D. Norris, Michelle Haber, Michelle J. Henderson

**Affiliations:** Experimental Therapeutics Program, Lowy Cancer Research Centre, Children’s Cancer Institute Australia for Medical Research, University of New South Wales and Sydney Children’s HospitalSydney, NSW, Australia

**Keywords:** neuroblastoma, MRP4/ABCC4, ATP-binding cassette transporter protein

## Abstract

Resistance to cytotoxic drugs is thought to be a major cause of treatment failure in childhood neuroblastoma, and members of the ATP-binding cassette (ABC) transporter superfamily may contribute to this phenomenon by active efflux of chemotherapeutic agents from cancer cells. As a member of the C subfamily of ABC transporters, multidrug resistance-associated protein MRP4/ABCC4 has the ability to export a variety of endogenous and exogenous substances across the plasma membrane. In light of its capacity for chemotherapeutic drug efflux, MRP4 has been studied in the context of drug resistance in a number of cancer cell types. However, MRP4 also influences cancer cell biology independently of chemotherapeutic drug exposure, which highlights the potential importance of endogenous MRP4 substrates in cancer biology. Furthermore, MRP4 is a direct transcriptional target of Myc family oncoproteins and expression of this transporter is a powerful independent predictor of clinical outcome in neuroblastoma. Together, these features suggest that inhibition of MRP4 may be an attractive therapeutic approach for neuroblastoma and other cancers that rely on MRP4. In this respect, existing options for MRP4 inhibition are relatively non-selective and thus development of more specific anti-MRP4 compounds should be a major focus of future work in this area.

## INTRODUCTION

Neuroblastoma, a cancer which arises from primitive cells of the sympathetic nervous system is the most common extra cranial solid tumor to occur in children. More than 50% of cases present as high risk disease, for which long-term survival rates remain below 50% (Brodeur, 2003; Maris et al., 2007). Within the last decade, considerable effort has been made toward universal risk stratification for neuroblastoma patients with a view to better tailoring of treatment. A recent international review of 8,800 patients throughout 1990–2002 confirmed patient age at diagnosis, tumor stage, DNA ploidy, chromosome 11q status, and *MYCN* status as important parameters to consider for patient risk stratification (Cohn et al., 2009). Detected in 20% of neuroblastoma cases, amplification of the *MYCN* proto-oncogene at chromosome 2p24 is one of the prominent indicators of aggressive clinical disease, reduced progression-free survival, and poor outcome (Maris et al., 2007). Consequently, the *MYCN *oncogene has been characterized extensively and in mice, its targeted expression toward neuroectodermal cells drives the development of neuroblastoma (Weiss et al., 1997). However, specific target genes regulated by *MYCN *and their contributions to an aggressive phenotype are still largely unknown. The C subfamily of the ATP-binding cassette (ABC) family of transporters, also known as the multidrug resistance-associated protein (MRP) family, consists of nine members, many of which are known for their roles in chemotherapeutic drug efflux. Of these, ABC subfamily C member 1 (*ABCC1*), *ABCC3*, and *ABCC4 *are direct transcriptional targets of MYCN and expression levels of each of these genes are independent prognostic factors for neuroblastoma (Norris et al., 1996, 2005; Haber et al., 2006; Porro et al., 2010; Henderson et al., 2011). Although ABC transporter genes are classically known for their contributions to drug resistance there has been growing evidence of their effects in neuroblastoma biology independent of drug efflux (Fletcher et al., 2010; Henderson et al., 2011). In this review, we examine the potential role of *ABCC4* in the malignant phenotype of neuroblastoma and other cancers and consider the potential benefits of targeting the MRP4 protein.

## THE MRP4 EFFLUX PUMP

MRP4 is a member of the C subfamily of ABC transporters. ABC transporters are mostly transmembrane proteins that mediate the ATP-dependent movement of a range of substances across cellular membranes (Russel et al., 2008). Located on chromosome 13q32.1, the *ABCC4* gene encodes the shortest member of the ABCC/MRP family (Russel et al., 2008) and mediates efflux of a variety of exogenous and endogenous molecules (**Table 1**).

**Table 1:** Drug and endogenous substrates of MRP4.

**Exogenous substances**		**Endogenous substances**	
**Pharmacological class**	**Drug**	**Structural class**	**Endogenous**
Folic acid derivative	Leucovorin	Cyclic and ADP nucleotides	cGMP cAMP ADP
DNA topoisomerase inhibitor	Topotecan* Irinotecan* (SN38)^1^	Purine analogs	Urate
		Eicosanoids	PGE^1^ PGE^2^ PGF_2α_
Anti-viral	Adefovir^†^	Folates	Folic acid
	PMEA^2†^	Bile acids	Cholate
	Ceftizoxime		Cholytaurine
Tenofovir^†^			
Anti-metabolite	6-Mercaptopurine^3†^	Conjugated steroids	DHEAS E_2_17βG
	Methotrexate		

PMEA, *para*-methoxyethylamphetamine; PG, prostaglandin; cGMP, cyclic-guanosine monophosphate; cAMP, cyclic-adenosine monophosphate; ADP, adenosine diphosphate; DHEAS, dehydroepiandrosterone; E_2_17βG, estradiol-17-β-D-glucuronide.

Table modified from Russel et al. (2008)

1 Norris et al., 2005

2 Schuetz et al., 1999

3 Janke et al., 2008

* Relevant to neuroblastoma treatment; camptothecins

† Nucleotide and nucleoside analog drugs.

Studies using *Abcc4 *gene knockout mice have begun to demonstrate the physiological role of MRP4, particularly in the liver, brain, and kidney. During cholestatic injury, where bile flow is obstructed from the liver, *Abcc4 *was up-regulated in rats and mice after bile duct ligation (Wagner et al., 2003; Denk et al., 2004) and also in patients with progressive familial intrahepatic cholestasis (Keitel et al., 2005). Mrp4 mediates the glutathione-dependent efflux of bile acids and its induction under these conditions is a protective response to guard against liver necrosis (Mennone et al., 2006; Rius et al., 2006). In addition to the removal of metabolites, Mrp4 is able to protect vital structures *in vivo* against various xenobiotics through active drug efflux (**Table 1**). *Abcc4*-deficient mice exhibit reduced renal excretion of the anti-viral drugs adefovir and tenofovir (Imaoka et al., 2007) and of the anticancer drug, topotecan (Leggas et al., 2004). Consequently, the levels of topotecan in the plasma and some tissues are elevated. *Abcc4 *deficiency also allows topotecan to cross the blood brain barrier and to penetrate the central nervous system and brain (Leggas et al., 2004). In addition, *Abcc4 *knockout mice had enhanced accumulation of the anti-viral drug9-(2-phosphonylmethoxyethyl)- adenine (PMEA) in the bone marrow, thymus, spleen, and intestine compared to wild-type littermates (Belinsky et al., 2007). Mrp4 is also known to be highly expressed in myeloid progenitor cells and has been shown to protect these cells against thiopurine-induced hematopoietic toxicity through active efflux (Krishnamurthy et al., 2008). Together, these studies highlight MRP4 as a versatile transporter able to protect vital organs against the accumulation of biological metabolites and a variety of xenobiotics, from anti-viral to chemotherapeutic drugs.

## SIGNALING SUBSTRATES OF MRP4

In addition to protecting cells against metabolic products and xenobiotics, MRP4 mediates transport of various endogenous bioactive substances (**Table 1**). Notably, a number of signaling molecules with established roles in normal tissue function and tumor biology are MRP4 substrates and these include cyclic nucleotides, prostaglandins, and leukotrienes (Chen et al., 2001; Reid et al., 2003; Rius et al., 2008).

Cyclic adenosine monophosphate (cAMP) plays a critical role as an intracellular second messenger, mediating a range of cellular functions. For example, cAMP-mediated signaling promotes monocyte differentiation (Giordano et al., 2003). Interestingly, *ABCC4* expression levels decrease with the differentiation toward mature leukocytes (Oevermann et al., 2009), which could promote cAMP accumulation and hence the strength of signaling down differentiation pathways. In cardiomyocytes, MRP4 regulates cAMP homeostasis which in turn controls the activity of key properties such as cardiac performance and structure (Sassi et al., 2011). Elevation of intracellular cAMP also promotes morphological differentiation and decreases proliferation in cultured neuroblastoma cell lines (Prasad et al., 2003; Sanchez et al., 2004).

In addition to supporting the normal development of various tissues, signaling molecules exported by MRP4 are able to support tumor growth in a variety of cancers. As mediators of the cyclooxygenase pathway, prostaglandins support an inflammatory microenvironment and can promote cell proliferation and survival in tumor cells, including neuroblastoma (Rasmuson et al., 2012). In addition, prostaglandin-E_2_ (PGE_2_) secreted from neuroblastoma cells can facilitate interactions with tumor-supportive bone marrow stromal cells (Ara et al., 2009). Clinical observations and mouse models have demonstrated the importance of prostaglandin-mediated pathways in colorectal cancer. Increased levels of PGE_2_ are reported in human colorectal adenomas and carcinomas compared to paired normal mucosa (Pugh and Thomas, 1994). Furthermore, clinical studies have shown that adenoma development in familial adenomatous polyposis patients can be prevented by inhibiting prostaglandin production (Chell et al., 2006). Conversely, mouse models have shown direct evidence of prostaglandin-promoted tumor growth, with an increase in tumor incidence following PGE_2_ treatment in Apc^Min/+^ mice (Greenhough et al., 2009).

Signaling via leukotrienes, another class of substrates transported by MRP4, promotes cell survival and proliferation through the activation of both autocrine and paracrine pathways. Up-regulation of leukotrienes in various cancers is thought to stimulate epithelial and surrounding stromal cells to produce relevant growth factors, pro-inflammatory mediators, and angiogenic factors which provide a tumor-supportive microenvironment (Wang and DuBois, 2010). In neuroblastoma cells, prolonged exposure to leukotriene B4 leads to increased cell viability in SK-N-BE(2)-C cells, while inhibitors of leukotriene production or signaling lead to cell cycle arrest and apoptosis (Sveinbjornsson et al., 2008).

## MRP4 AND DRUG RESISTANCE IN CANCER

Chemotherapy resistance is a major obstacle to successful cancer treatment and members of the ABCC/MRP transporter family are perhaps best known for their abilities to confer drug resistance through the active export of structurally dissimilar chemotherapeutic compounds in various cancers (Borst et al., 2000). Clinically relevant drugs known to be transported by MRP4 include nucleoside and nucleotide analogs (Chen et al., 2001) and, relevant to neuroblastoma, the camptothecins irinotecan (Norris et al., 2005) and topotecan (Tian et al., 2005) (**Table 1**). Therefore, the role of MRP4 in establishing drug resistance has been explored in a number of cancer cell lines.

HepG2 cells transfected with the human *ABCC4* plasmid showed enhanced resistance to irinotecan, topotecan, and cyclophosphamide, all of which are used in neuroblastoma treatment or are in trial for use (Langler et al., 2002; Kushner et al., 2006; London et al., 2010). Using established MRP4 inhibitors such as MK571 and celecoxib, resistance to these cytotoxic agents was significantly reduced (Tian et al., 2005, 2006). Resistance to irinotecan and reduction in its intracellular accumulation was additionally shown in HEK-293 cells transfected with the human *ABCC4 *plasmid when compared to parental HEK-293 cells (Norris et al., 2005).

Expression of MRP4 in leukemia cells correlated with drug resistance to relevant chemotherapeutic agents used in leukemia treatment. A fourfold increase in MRP4 expression was observed in CEM-MP5 leukemia cells, which were selected for resistance through step-wise exposure to 6-mercaptopurine (6-MP). Compared to the parental CEM cell line, CEM-MP5 cells displayed a 100-fold greater resistance to 6-MP (Peng et al., 2008). Additionally, the acute myeloid leukemia (AML) cell line K562/ADR showed elevated expression of MRP4 compared to parental K562, which had a greater resistance to adriamycin, a standard chemotherapy for AML. Although there is no evidence of direct efflux of adriamycin by MRP4, knockdown of MRP4 through lentiviral-mediated shRNA within the K562/ADR cell line enhanced adriamycin-induced apoptosis (Liu et al., 2012), suggesting MRP4 may contribute drug resistance by other means, such as removal of toxic metabolites associated with drug exposure.

In the gastric cancer cell line SGC7901/DDP, resistance to cisplatin was developed through incremental cisplatin exposure and was associated with increased MRP4 expression. This resistance was reversed upon siRNA-mediated MRP4 knockdown (Zhang et al., 2010). Although cisplatin is not an MRP4 substrate, the elevation of MRP4 might promote cell survival through increased in export of autocrine signaling molecules involved in regeneration, such as various nucleotides or prostanoids (Aleksunes et al., 2008).

## MRP4, AN INDEPENDENT PROGNOSTIC FACTOR IN NEUROBLASTOMA

In attempt to gain insight into the potential clinical relevance of MRP4, the relationship between expression, pathology, and patient outcome has been examined in several tumor types. To address this question in neuroblastoma, in two independent studies, quantitative reverse transcriptase-PCR was used to determine *ABCC4* expression levels in primary neuroblastoma tumors obtained prior to treatment (Norris et al., 2005; Henderson et al., 2011). In both studies, *ABCC4 *expression was associated with *MYCN *amplification as well as advanced tumor stage. Interestingly, chromatin immunoprecipitation and luciferase reporter assays were used to demonstrate that *MRP4 *expression is directly regulated by the *MYCN *and *MYC *oncogenes (Porro et al., 2010), raising the possibility that *MYCN* induction of *ABCC4 *expression may contribute to the malignant phenotype driven by *MYCN* in neuroblastoma.

More importantly, *ABCC4 *expression in primary neuroblastoma is strongly associated with reduced event-free survival and overall survival and multivariate analysis revealed that *ABCC4 *expression retained prognostic significance following adjustment for tumor stage, age, and *MYCN *amplification (Henderson et al., 2011). Analysis of a microarray dataset from a third independent cohort of patients enrolled in the German Neuroblastoma Trial NB90-NB2004 confirmed these findings (Oberthuer et al., 2006; Henderson et al., 2011). For none of the three cohorts however, were any of the chemotherapeutic agents used to treat patients substrates of MRP4, suggesting the link between MRP4 and poor patient outcome is not attributable to resistance mechanisms related to drug efflux. Furthermore, expression of *ABCC4* was determined in primary untreated neuroblastoma tumors indicating that *ABCC4 *expression levels were independent of prior treatment exposure. These observations suggested that enhanced MRP4 expression contributes to poor outcome in neuroblastoma through mechanisms other than facilitation of drug export. In support of this, siRNA-mediated knockdown of MRP4 in the neuroblastoma cell lines SK-N-BE(2)C and SH-SY-5Y reduced cell proliferation and clonogenicity (Henderson et al., 2011). These studies indicate MRP4 expression may be an important biological factor in neuroblastoma, outside of its ability to contribute to drug efflux. Studies currently in progress involving cross breeding between *MYCN* transgenic mice and *Abcc4 *gene knockout mice will further assess the biological impact of MRP4 in neuroblastoma development, progression, and treatment response.

## MRP4 IN OTHER CANCERS

While definitive studies are yet to be reported, some evidence suggests MRP4 may be important in the biology of other cancers. In addition to playing a role in drug-resistant leukemia cell lines, MRP4 appears to regulate leukemia cell proliferation and differentiation independently of drug efflux through the endogenous MRP4 substrate cAMP. Blockage of MRP4 through shRNA or the MRP4 inhibitor, probenecid, in the human leukemia cell line U937 caused intracellular accumulation of cAMP and consequent leukemic maturation toward a more differentiated phenotype (Copsel et al., 2011). These findings are consistent with the apparent role for MRP4 and cAMP-mediated signaling in normal hematopoietic cell development, where *ABCC4 *expression levels decrease during differentiation toward mature leukocytes (Oevermann et al., 2009). Other studies suggest a vital role for MRP4 in T-cell migration, where inhibition of MRP4, either by viral shRNA or sildenafil, impaired the abilities of interstitial dendritic cells to migrate toward lymph node chemokines (van de Ven et al., 2008), raising the possibility that MRP4 could also influence migratory properties of either cancer cells or their neighbors.

Polymorphisms within the *ABCC4 *gene are associated with poor outcome in acute lymphoblastic leukemia. Persons with the C-allele of the TC-1393 polymorphism, which may influence *ABCC4 *gene promoter activity, had longer event-free survival and persons with the A-allele of the CA-934 polymorphism had reduced event-free survival compared to the respective other genotypes. Despite the CA-934 polymorphism, the level of MRP4 expression within the liver and amount of 6-MP accumulation in the kidney remained unchanged, suggesting the CA-934 polymorphism does not contribute a drug resistant phenotype within this patient cohort (Ansari et al., 2009). Although the mechanism underlying CA-934 polymorphism and poor outcome is unknown, the efflux of an endogenous substrate is one potential mechanism.

MRP4 protein was more abundant in pancreatic cancers when compared to adjacent non-cancerous pancreatic tissue and knockdown of MRP4 using shRNA in two pancreatic cancer cell lines, Panc1 and BxPC-3, inhibited cell growth and clonogenicity through cell cycle arrest (Zhang et al., 2011). These initial findings suggest that MRP4 could contribute to pancreatic cancer progression, however, the underlying mechanism responsible for the observed cell cycle arrest and reduction in cell proliferation during MRP4 knockdown is currently unknown.

Androgens are steroidal hormones required for normal prostate development and are also important signaling molecules in driving prostate cancer. Upon androgen binding, the androgen receptor translocates into the nucleus where it binds to androgen response elements in androgen-responsive genes, including various genes involved in cell proliferation and apoptosis (Heinlein and Chang, 2004). Using oligonucleotide gene array analysis, two groups independently identified *ABCC4 *to be one such androgen regulated gene (Cai et al., 2006; Ho et al., 2008). Due to increased androgen signaling, MRP4 expression is higher in prostate cancers than in normal prostate cells (Suzuki et al., 2003; Ho et al., 2008). Anti-androgen treatment using bicalutamide reduced MRP4 expression in the LnCAP prostate cancer cell line and tumors from patients treated with androgen ablation pre-operatively were found to have lower levels of MRP4 expression compared to those from uncastrated prostate cancer patients. Despite MRP4 being a potentially important target of the androgen receptor, no correlation was seen between MRP4 expression and known prognostic factors or relapse free survival (Ho et al., 2008).

Despite these observations and the association between MRP4 expression and drug resistance in various cancer cell lines, few studies have adequately explored the relationship between MRP4 expression and clinical outcome in these cancers, and, with the exception of neuroblastoma, no association has been reported (Steinbach et al., 2003; Zhang et al., 2011). Future studies in appropriate *in vitro *and tumor models, together with large prospective studies of tumor specimens to examine associations between gene expression and clinical outcome are required to follow-up these suggestive observations.

## TARGETING MRP4 FOR THE TREATMENT OF NEUROBLASTOMA

In neuroblastoma, given the strong association between MRP4 expression and poor clinical outcome, the effects on cancer cell biology, and the use of MRP4 cytotoxic substrates such as topotecan in relapsed neuroblastoma patients (Saylors et al., 2001; Langler et al., 2002; London et al., 2010), treatment through MRP4 inhibition may provide a novel approach to influence both treatment efficacy and tumor growth.

A number of pharmacological agents with MRP4 inhibitory activity have been identified. However, most are non-specific and are able to target other ABC transporter family members (**Table 2**). Experience from early clinical trials using inhibitors of the multidrug transporter P-glycoprotein (P-gp/MDR1) indicates that future generations of inhibitors should be selective if the benefit of targeting MRP4 is to be effectively realized (Szakacs et al., 2006). Such inhibitors should block transport of the relevant exogenous or endogenous substrates, thus identification of key physiological substrates should be a focus of future work. Screening for selective MRP4 small molecule inhibitors may allow for the development of a more targeted approach for neuroblastoma therapy and better treatment strategies for other malignancies, particularly if patients whose tumors have high levels of MRP4 are selected for intervention.

**Table 2:** MRP4 inhibitors and their selectivity.

**MRP4 inhibitor**	**Mode of action/ structural class**	**Additional targets**
Dipyridamole	Nucleoside transport inhibition	P-gp, MRP1
Sildenafil	PDE5 inhibitor	MDR1, BCRP
MK571	Leukotriene antagonist	MRP1, MRP2, MRP5
Indomethacin	Non-steroidal anti-inflammatory	MRP1, MRP2
Celecoxib	COX-2 inhibitor	MRP1
Probenecid	Organic anion	MRP2, MRP3
Sulindac	Non-steroidal anti-inflammatory	MRP2
Losartan	Angiotensin II type I receptor antagonist	P-gp
Quercetin	Flavonoid	MRP1, MRP2, MRP5

*Table modified from Russel et al. (2008).*

*P-gp, P-glycoprotein; MRP, multidrug resistance protein; PDE, phosphodiesterase; COX, cyclooxygenase*

Small molecule inhibitors are able to inhibit the function of specific proteins through a number of mechanisms. These include binding DNA at various gene promoters, thereby inhibiting gene expression at either the transcriptional or translational level, through to inhibiting protein–substrate interaction through competitive binding or inducing a protein conformational change (Yap et al., 2012). High-throughput screens commonly involve rapidly testing tens of thousands of potential drug candidates in target-based or cell-based assays with the aim of identifying potentially active compounds from large compound collections (Macarron et al., 2011). Small molecule inhibitors have the advantage of being efficiently produced and further developed in order to inhibit relevant oncogenic targets (Bleicher et al., 2003). Such screening methods led to development of Imatinib (Glivec), which is a potent inhibitor of the Abl tyrosine kinase and selectively kills leukemia cells expressing the *Bcr-Abl* oncogene (Druker et al., 1996). With a high response rate to therapy and a favorable cytotoxic profile compared to standard chemotherapy this compound and its relatives represent an incredible breakthrough in therapy for chronic myelogenous leukemia and other Philadelphia chromosome-positive leukemias (O’Brien et al., 2003; Novak, 2004).

High-throughput screening of chemical libraries has been used successfully to identify novel inhibitors of ABC transporters (Burkhart et al., 2009). A library of 2,300 analogs based upon P-gp modulators was screened for MRP1 inhibition through a cell-based readout system. The breast cancer cell line MCF7/VP16, which has amplified MRP1 levels but low P-gp levels was transduced with a p53-responsive LacZ reporter to create a read out of MRP1 inhibition during accumulation of doxorubicin, a cytotoxic MRP1 substrate. Based on these screening efforts, six structural scaffolds which effectively inhibit MRP1 were identified (Burkhart et al., 2009).

Similarly, we have developed high-throughput screening methodology for MRP4 inhibitors using MRP4-over expressing HEK-293 cells which exhibit sixfold resistance to the MRP4 cytotoxic substrate 6-MP. From 30,000 random chemical small molecules we were able to identify 19 “hit” compounds able to reverse the resistance of MRP4-overexpressing HEK-293 cells to 6-MP, and to cause intracellular 6-MP accumulation. Remarkably, in the absence of drug exposure, two of the compounds also recapitulated the effects of siRNA-mediated MRP4 knockdown in that they inhibited growth of the neuroblastoma cell line, SK-N-BE(2)C, and this was accompanied by extension of neuritic processes, consistent with enhanced morphological differentiation. Each of the compounds has a distinct chemical structure and screening of structural analogs has identified additional anti-MRP4 compounds suitable for pre-clinical testing.

One potential problem with hits derived from high-throughput screening, however, may arise if the compounds do not have a single molecular target (Bleicher et al., 2003). Understanding the complete spectrum of intracellular targets of a novel inhibitor will identify potential toxicities and facilitate prediction of cancer behavior toward a particular inhibitor. Given the high structural homology and substrate overlap of MRP4 with other ABC transporter proteins, such as MRP1, MRP5, and ABCG2 (Borst et al., 2000; Klaassen and Aleksunes, 2010), it is essential to screen for specific MRP4 inhibitors in order to limit potential off target effects. To overcome this issue, candidate inhibitors can be tested on cell lines overexpressing a range of ABC transporters, each paired with their respective parental control, in the presence of cytotoxic substrate relevant to each transporter in order to determine the specificity of inhibitors (Burkhart et al., 2009). Additionally, inside-out vesicle assays can also be used to characterize ABC transporter substrate specificity and the effects on of inhibitors on transporter-mediated excretion of individual substrates (Schlemmer and Sirotnak, 1994; Loe et al., 1996).

## CONSIDERATIONS AND FUTURE DIRECTIONS

MRP4 offers a valuable and novel target for the treatment of neuroblastoma and potentially other malignancies for which evidence implicates MRP4 in cancer biology and chemotherapy resistance. With specific MRP4 inhibitors, it is expected that side effects would be minimal given MRP4 knock-out mice do not show any obvious abnormalities (Belinsky et al., 2007). However, drug distribution to various organ compartments and potential toxicities of MRP4 inhibitors in combination with established chemotherapies must be considered and their effects profiled in combination with relevant chemotherapeutics in appropriate preclinical models. A potential benefit from the role of MRP4 in biodistribution lies in the possibility that MRP4 inhibitors may enhance drug availability to tumor tissues through uptake across the blood brain barrier or reduced renal elimination. This may be particularly beneficial for drugs in clinical trials for the treatment of neuroblastoma such as irinotecan and topotecan and, as suitable cohorts emerge, this possibility should be explored by examining the relationship between clinical outcome and MRP4 expression in primary tumors of neuroblastoma patients receiving these drugs (Kushner et al., 2006; Simon et al., 2007). Furthermore, the ability to examine pharmacological inhibition of MRP4 using *in vitro *and *in vivo *models will offer a tool with which to gain greater insight into its role in the transcriptional program of MYCN in neuroblastoma.

## Conflict of Interest Statement

The authors declare that the research was conducted in the absence of any commercial or financial relationships that could be construed as a potential conflict of interest.
